# Deep learning for dose-averaged linear energy transfer estimation in pencil-beam scanning and double scattering proton radiotherapy plans with uncertainty-aware external validation^☆^

**DOI:** 10.1016/j.phro.2026.100998

**Published:** 2026-05-15

**Authors:** Aaron Kieslich, Yerik Singh, Martina Palkowitsch, Sebastian Starke, Fabian Hennings, Esther G.C. Troost, Mechthild Krause, Jona Bensberg, Armin Lühr, Feline Heinzelmann, Christian Bäumer, Beate Timmermann, Nicolas Depauw, Helen A. Shih, Steffen Löck

**Affiliations:** a OncoRay – National Center for Radiation Research in Oncology, Faculty of Medicine and University Hospital Carl Gustav Carus, TUD Dresden University of Technology, Helmholtz-Zentrum Dresden-Rossendorf, Dresden, Germany; b Helmholtz-Zentrum Dresden-Rossendorf, Institute of Radiooncology – OncoRay, Dresden, Germany; c Institute for Medical Information Processing, Biometry, and Epidemiology, Ludwig-Maximilians-Universität München, München, Germany; d Helmholtz-Zentrum Dresden-Rossendorf, Department of Information Services and Computing, Dresden, Germany; e German Cancer Consortium (DKTK), Partner Site Dresden, and German Cancer Research Center (DKFZ), Heidelberg, Germany; f National Center for Tumor Diseases (NCT), NCT/UCC Dresden, a partnership between DKFZ, Faculty of Medicine and University Hospital Carl Gustav Carus, TUD Dresden University of Technology, and Helmholtz-Zentrum Dresden-Rossendorf (HZDR), Germany; g Department of Radiotherapy and Radiation Oncology, Faculty of Medicine and University Hospital Carl Gustav Carus, TUD Dresden University of Technology, Dresden, Germany; h TU Dortmund University, Department of Physics, Dortmund, Germany; i West German Proton Therapy Center Essen (WPE), University Hospital Essen, Essen, Germany; j German Cancer Consortium (DKTK), Partner Site Essen, and German Cancer Research Center (DKFZ), Heidelberg, Germany; k Clinic for Particle Therapy, University Hospital Essen, Essen, Germany; l Department of Radiation Oncology, Massachusetts General Hospital/Mass General Brigham and Harvard Medical School, Boston, MA, United States

**Keywords:** Proton radiotherapy, Linear energy transfer, Deep learning, Pencil beam scanning, Double scattering, Uncertainty quantification

## Abstract

**Background and Purpose:**

Accounting for the linear energy transfer (LET) in proton radiotherapy may reduce treatment-related side effects. When Monte Carlo (MC) simulations are unavailable, deep-learning (DL) surrogate models can be applied. We develop DL LET models for brain tumour patients and assess their uncertainty for an external dataset lacking LET reference.

**Material and Methods:**

A multi-institutional dataset of 570 patients with 605 treatment plans was used for model development and evaluation. DL models predicting dose-averaged LET (LET${}_{d}$) were trained separately for pencil-beam scanning (PBS) and double scattering (DS) treatments, as well as a combined PBS+DS model. Deviations from MC reference were evaluated by median and 98th percentile voxelwise absolute errors (VAE) in organs at risk and the treatment target. Uncertainty was evaluated via deep ensemble variance and latent space distance, correlated with the median VAE, and applied to an external DS cohort without LET${}_{d}$ reference.

**Results:**

The PBS+DS model achieved average median VAEs below 0.42 keV/μm. Ensemble variance and latent space distance showed positive correlations with error, with the strongest association observed for ensemble variance (up to ρ= 0.88). Both uncertainty metrics indicated an estimated average median VAE below 0.54 keV/μm for the DS model on the external DS data.

**Conclusion:**

DL models accurately approximate LET${}_{d}$ for PBS and DS proton radiotherapy. Uncertainty estimation provides indirect evidence of model reliability and enables performance estimation in cohorts lacking LET${}_{d}$ reference, supporting safer application in retrospective analyses and clinical research.

## Highlights

• Deep learning predicted LET${}_{d}$ from clinical dose distributions.
• LET${}_{d}$ models were trained for pencil-beam scanning and double scattering.
• Uncertainty was quantified by latent space distance and ensemble variance.
• Uncertainty metrics correlated with LET${}_{d}$ prediction error across cohorts.
• Uncertainty estimates supported performance assessment without LET${}_{d}$ reference.

## Graphical abstract

**Figure:**
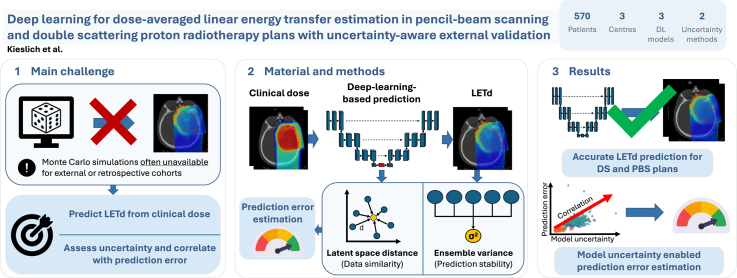

## 1 Introduction

Proton radiotherapy offers improved sparing of organs at risk compared to photon radiotherapy by exploiting the finite range of protons [1], [2]. However, the biological effectiveness of proton radiation introduces additional uncertainty. In clinical practice, a generic relative biological effectiveness (RBE) of 1.1 is assumed for proton doses, but in reality proton RBE tends to increase with increasing linear energy transfer (LET) [2], [3], [4], [5], [6]. This variability may increase normal tissue toxicity beyond what a constant RBE predicts [7], [8], [9], [10], [11], [12], [13], [14], [15], [16]. Monte Carlo (MC) transport simulations are the gold standard for computing LET in realistic patient geometries and have been used to simulate LET distributions for both pencil-beam scanning (PBS) and passive double scattering (DS) proton radiotherapy treatments [17], [18]. However, MC simulations are computationally demanding and require detailed knowledge of the treatment beamline and delivery parameters [19]. As a result, LET calculation is often not feasible for external or retrospective patient data, which hinders large-scale investigations into the clinical implications of proton RBE variability [20].

To address these challenges, several studies have proposed deep learning (DL) models to approximate LET distributions [21], [22], [23], [24]. Recently, we demonstrated that a three-dimensional convolutional neural network (CNN) can predict the dose-averaged LET (LET${}_{d}$) distribution of protons based on the planned dose distribution in brain tumour patients treated with PBS [25].

In the present study, we extend the previous results in several important aspects. First, the training dataset is expanded to include DS treatment plans, enabling model development across both major proton delivery techniques. Second, we evaluate and integrate uncertainty quantification methods, including deep ensemble variance and latent space distance. These methods provide indirect evidence of prediction reliability and allow exploration of model performance in scenarios lacking reference LET${}_{d}$ data. In this context, we demonstrate the application of the developed models to an external cohort treated with DS, highlighting how uncertainty estimation can serve as a complementary safeguard for model use in real-world data without reference.

## 2 Material and methods

### 2.1 Patient data

This retrospective study involved four patient cohorts. Two cohorts were retrospectively collected at the University Proton Therapy Dresden (UPTD), differentiated by treatment technique: the UPTD PBS cohort consisted of 212 plans from 195 patients treated with PBS, while the UPTD DS cohort comprised 152 plans from 152 patients treated with DS. Additionally, a third cohort was collected from patients treated with PBS at the West German Proton Therapy Centre Essen (WPE) as part of the prospective registry study ProReg (DRKS00004384), comprising 145 plans from 127 patients. A fourth cohort, treated with DS at the Massachusetts General Hospital (MGH) in Boston, consisted of 96 plans from 96 patients. In the PBS cohorts, the number of plans exceeds the number of patients because 35 patients were treated with sequential plan subseries for additional boost target volumes.

For all cohorts, clinically approved planning computed tomography (CT) images, contours of regions of interest (ROIs), and corresponding clinical dose distributions (dose-to-water) were available. LET${}_{d}$ distributions were generated using MC simulations. RayStation’s MC algorithm was utilised for both UPTD PBS^2^ and WPE^3^ cohorts, whereas an in-house developed MC simulation implemented in TOPAS [26] was used for UPTD DS patients [17], [27]. LET${}_{d}$ was defined as the unrestricted LET${}_{d}$ for primary and secondary protons scored in unit-density tissue. MC simulations were performed with a statistical uncertainty of 0.5%. For the MGH cohort, no reference MC LET${}_{d}$ data were available.

This retrospective analysis was approved by the Ethics Committee of the Dresden University of Technology, Germany (EK252062022). Detailed characteristics of all four cohorts are summarised in Table S1.

### 2.2 Model development

The DL models employed in this study consisted of a SegResNet architecture [28], utilising the same image preprocessing steps and model parameters as previously described [25]. The model used the absorbed dose distribution as input, normalised on a per-plan basis to the mean dose within the clinical target volume (CTV). For plans with a simultaneous integrated boost (SIB), the mean dose of the boost volume was used for normalisation. The only modification introduced in the current study pertained to the calculation of the mean absolute error loss, which was restricted exclusively to voxels in the region receiving a dose exceeding 4% of the mean target dose (e.g., 2 Gy for a mean dose of 50 Gy), defined as the relevant dose region. LET${}_{d}$ values outside this region were set to zero in the postprocessing step after prediction. Further details regarding preprocessing method, network architecture and training hyperparameters are provided in the Supplementary material A and B.

Three distinct training approaches were investigated: a PBS model, a DS model, and a combined PBS+DS model. To create representative training and validation sets, stratified sampling based on the volume of the CTV and the presence of SIBs was employed within the UPTD cohorts, with 78% of the plans allocated for training and the remaining 22% for validation. The PBS and DS models were each trained on their respective UPTD training subsets and validated on the corresponding validation subsets, as well as on the other-technique UPTD cohort and the WPE cohort. The combined PBS+DS model was trained jointly on both UPTD training subsets and validated on both validation subsets and the WPE cohort. An overview of all cohorts, the corresponding training/validation splits, and their assignment to the three training approaches is provided in Table S2.

### 2.3 Performance evaluation

The model performance was quantified using the median and the 98th percentile of the voxelwise absolute error (VAE) between MC and DL LET${}_{d}$, characterising typical and near-maximum deviations. This evaluation was performed for clinically relevant ROIs, including the brain, brainstem, CTV, chiasm, lacrimal glands, lenses, and optic nerves, with all ROIs restricted to voxels within the relevant dose region.

To assess clinical relevance, we evaluated the impact of LET${}_{d}$ accuracy on normal tissue complication probability (NTCP) predictions. We considered NTCP models for ocular toxicity (grade ≥2) [29], memory impairment (grade ≥1) [30], and blindness (using chiasm and ipsilateral optic nerve) [31]. In addition, we incorporated the patient-level risk prediction for development of one or more contrast-enhancing brain lesions proposed by Bahn et al. [9]. Details of the risk models and NTCP evaluation workflow are provided in Supplementary material C.

**Fig. 1:**
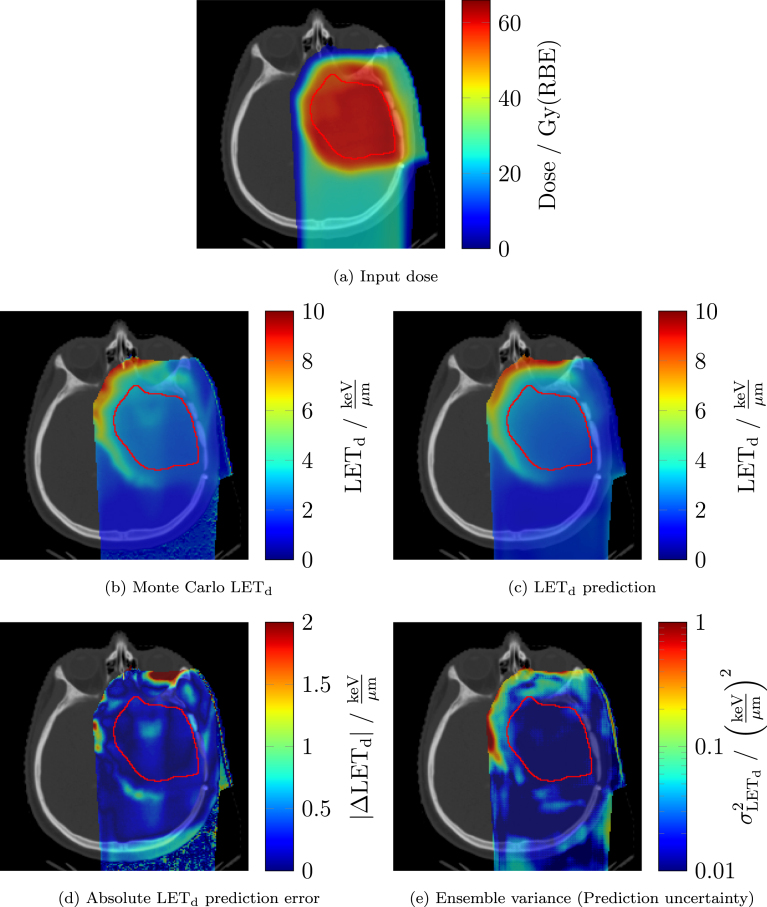
Representative example from the validation set of the UPTD DS cohort for the combined PBS+DS model. Shown are the planned dose distribution (a), Monte Carlo reference ${\mathrm{LET}}_{d}$ (b), the corresponding model prediction (c), the voxelwise absolute ${\mathrm{LET}}_{d}$ prediction error (d), and the predicted uncertainty map from the ensemble variance method (e). Only the relevant dose region is displayed, defined as voxels with normalised dose greater than 0.04 (dose divided by the mean dose in the CTV). Notation Gy(RBE) follows ICRU-93 definition.

**Fig. 2:**
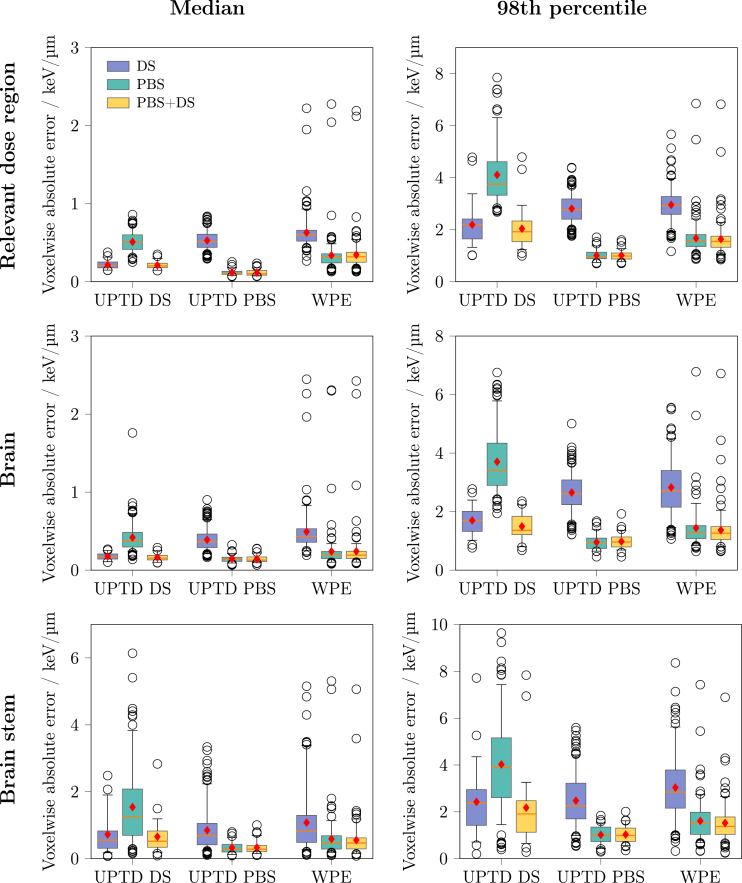
Voxelwise absolute error distributions for the validation sets within the respective regions of interest (ROIs): relevant dose region (top row), brain (middle row), and brain stem (bottom row). For each ROI, voxelwise errors were aggregated across all voxels using either the median (left column) or the 98th percentile (right column). Each boxplot displays the median, the 25th and 75th percentiles (box), and the whiskers denoting the data range according to Tukey’s rule. Each plot shows the distribution of aggregated voxelwise absolute errors across patient cohorts (UPTD DS, UPTD PBS, WPE) and modelling approaches (DS, PBS, PBS+DS). Red diamonds indicate the mean aggregated voxelwise absolute error within each group.

**Fig. 3:**
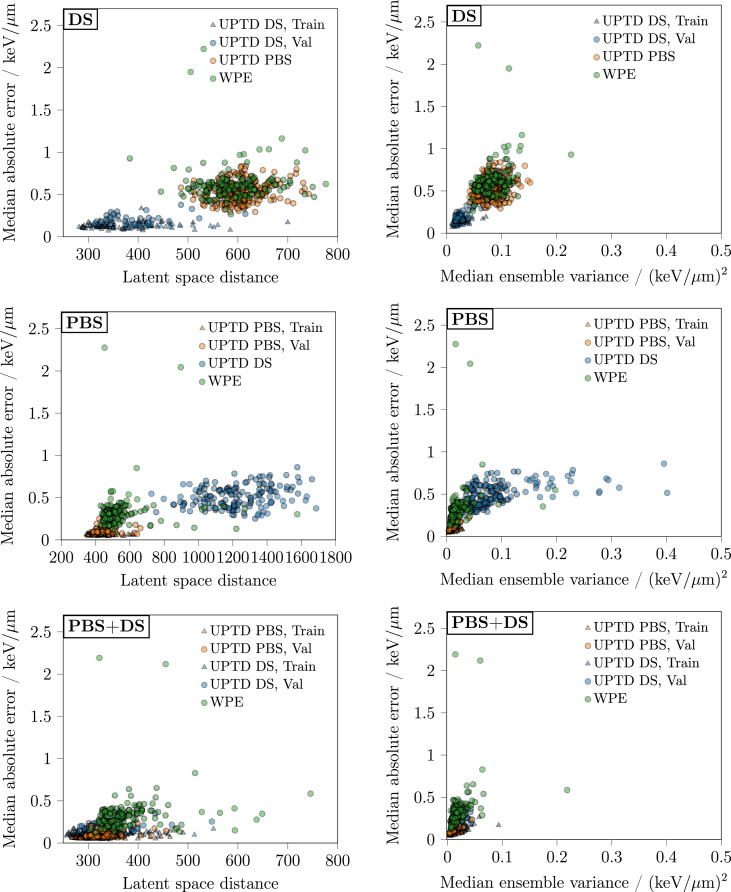
Model uncertainty metrics versus median voxelwise absolute error within the relevant dose region. The left column presents the results for the latent space distance, while the right column shows results for the median ensemble variance. Rows correspond to DS, PBS, and PBS+DS models. Abbreviations: Train = Training, Val = Validation.

### 2.4 Uncertainty estimation

To assess the reliability of LET${}_{d}$ predictions, we implemented two complementary uncertainty estimation techniques [32]: latent space distance [33], [34] and deep ensemble variance [35].

Latent space distance measures input data similarity and is based on the assumption that inputs dissimilar to the training data are more likely to result in unreliable predictions. To assess this distance, the feature map produced by the final encoder layer of the SegResNet architecture was reshaped into a one-dimensional latent representation of the input dose distribution. To improve comparability across features, the latent representations of the individual samples were further scaled using Min–Max normalisation on a per-feature basis using the training cohort. We then defined the latent space distance for each sample as the average Euclidean distance in the scaled latent space to its 25 nearest neighbours from the training cohort, yielding a single scalar uncertainty value per plan.

The deep ensemble variance approach assesses output prediction stability. We utilised an ensemble of five SegResNet models obtained from five-fold cross-validation using the respective training cohort. For each input, LET${}_{d}$ predictions were generated by all ensemble members and a voxelwise variance map was computed across the ensemble. To obtain a single scalar uncertainty value per plan, the voxelwise variance was aggregated by taking the median variance within the relevant dose region. Higher median variance across ensemble members was interpreted as increased epistemic uncertainty. Both per-plan uncertainty metrics were compared to the median VAE in the relevant dose region by Spearman correlation.

## 3 Results

### 3.1 Prediction error

Figs. 1(a)–(d) presents a representative validation example of the combined PBS+DS model from the UPTD DS cohort, including the input dose, the reference MC LET${}_{d}$, the predicted LET${}_{d}$ and the absolute error map. Larger prediction errors occurred predominantly at distal beam edges with pronounced dose gradients. Corresponding qualitative examples for the UPTD PBS and WPE cohorts are provided in Figures S1 and S2, showing similar trends. Fig. 2 shows the distribution of the median and 98th percentile VAE within the relevant dose region, the brain and the brainstem across modelling approaches (PBS, DS, PBS+DS) and cohorts (UPTD PBS, UPTD DS, WPE).

A pronounced drop in performance was observed when applying the DS model to PBS data (UPTD PBS, WPE) and, conversely, when applying the PBS model to DS data (UPTD DS). For example, on the UPTD PBS cohort, the DS model yielded an average median VAE in the relevant dose region of 0.54 keV/μm (2.86 keV/μm for the 98th percentile), versus 0.07 keV/μm (1.03 keV/μm) for the PBS model. Similarly, on the UPTD DS cohort the PBS model exhibited an average median VAE of 0.53 keV/μm (4.25 keV/μm for the 98th percentile), compared to 0.22 keV/μm (2.34 keV/μm) for the DS model.

The combined PBS+DS model achieved average median VAEs of 0.12 keV/μm on UPTD PBS, 0.22 keV/μm on UPTD DS, and 0.42 keV/μm on the external WPE cohort. The corresponding 98th percentile VAEs were 1.03, 2.19, and 1.76 keV/μm, respectively. Complete error values for all ROIs and both error statistics are listed in Tables S3 and S4.

The NTCP evaluation was consistent with the VAE analysis, showing similar trends. Full results are presented in Table S5 and Figure S3.

### 3.2 Evaluation of uncertainty metrics on cohorts with reference LET${}_{d}$ distribution

Fig. 1(e) shows the voxelwise ensemble variance uncertainty map for a representative example UPTD DS validation plan using the combined PBS+DS model. Regions of elevated uncertainty corresponded spatially well to regions of increased absolute prediction error (Fig. 1(d)). Similar spatial trends were observed for the UPTD PBS and WPE cohorts (Figures S1 and S2).

The quantitative results of both uncertainty estimation techniques are summarised in Table 1. The corresponding scatterplots in Fig. 3 visualise the correlations between the uncertainty metrics and the LET${}_{d}$ prediction error.

For the latent space distance approach, correlations between the latent space distance and the median VAE were generally weak when evaluated within individual cohorts (range ρ=0.08 to 0.30). This suggests that the metric reflects uncertainty patterns only to a limited extent on a cohort-specific level. However, when data from all cohorts were combined, correlations increased to moderate-to-strong levels (ρ=0.65, 0.81, and 0.52 for the DS, PBS, and PBS+DS models, respectively), indicating that the latent space distance captures broader, cross-cohort uncertainty trends more effectively.

**Table 1:** Spearman rank correlations (ρ) between uncertainty estimates and the median voxelwise absolute error. The two columns report ρ between the latent space distance and the median ensemble variance with the median absolute prediction error. All metrics were computed within the relevant dose region. Results across cohorts (UPTD DS, UPTD PBS, WPE), sets (training, validation) and modelling approaches (DS, PBS, PBS+DS) are listed. “Overall (with Train)” summarises correlations across all available samples from all cohorts and sets, whereas “Overall (without Train)” excludes training samples and aggregates validation cases only. Numbers in parentheses denote 95% confidence intervals obtained from 1000 bootstrap samples.

Cohort	Set	ρ (vs median voxelwise absolute error)	
		Latent space distance	Median ensemble variance
**DS**			
UPTD DS	Train	0.15	0.52
		(−0.02–0.32)	(0.36–0.64)
UPTD DS	Val	0.28	0.65
		(−0.07–0.56)	(0.37–0.81)
UPTD PBS	Val	0.13	0.22
		(−0.01–0.25)	(0.09–0.34)
WPE	Val	0.15	0.37
		(−0.01–0.31)	(0.21–0.51)
Overall (without Train)		0.30	0.46
		(0.20–0.40)	(0.37–0.54)
Overall (with Train)		0.65	0.74
		(0.59–0.70)	(0.69–0.79)

**PBS**			
UPTD DS	Val	0.22	0.49
		(0.05–0.36)	(0.36–0.60)
UPTD PBS	Train	0.12	0.68
		(−0.02–0.26)	(0.56–0.76)
UPTD PBS	Val	0.12	0.85
		(−0.18–0.45)	(0.68–0.94)
WPE	Val	0.22	0.53
		(0.06–0.39)	(0.40–0.64)
Overall (without Train)		0.71	0.81
		(0.65–0.76)	(0.78–0.85)
Overall (with Train)		0.81	0.88
		(0.78–0.84)	(0.86–0.90)

**PBS+DS**			
UPTD DS	Train	0.08	0.62
		(−0.10–0.27)	(0.48–0.73)
UPTD DS	Val	0.14	0.64
		(−0.20–0.48)	(0.29–0.84)
UPTD PBS	Train	0.13	0.75
		(−0.02–0.26)	(0.66–0.81)
UPTD PBS	Val	0.26	0.90
		(−0.04–0.51)	(0.82–0.94)
WPE	Val	0.30	0.59
		(0.12–0.46)	(0.46–0.70)
Overall (without Train)		0.46	0.57
		(0.34–0.57)	(0.46–0.67)
Overall (with Train)		0.52	0.65
		(0.46–0.58)	(0.59–0.70)

Abbreviations: ρ= Spearman rank correlation, LET${}_{d}$= dose-averaged LET, MC = Monte Carlo, DS = Double Scattering, PBS = Pencil Beam Scanning, Train = Training, Val = Validation.

For the deep ensemble variance method, correlations between the median ensemble variance and the corresponding median VAE were consistently higher than for the latent space distance across all models. Strong correlations were again observed across all models when combining all available data (ρ=0.74, 0.88, and 0.65 for the DS, PBS, and PBS+DS models, respectively), whereas correlations within individual cohorts were moderate to strong (range ρ=0.22 to 0.90).

### 3.3 Uncertainty evaluation on the MGH cohort

**Fig. 4:**
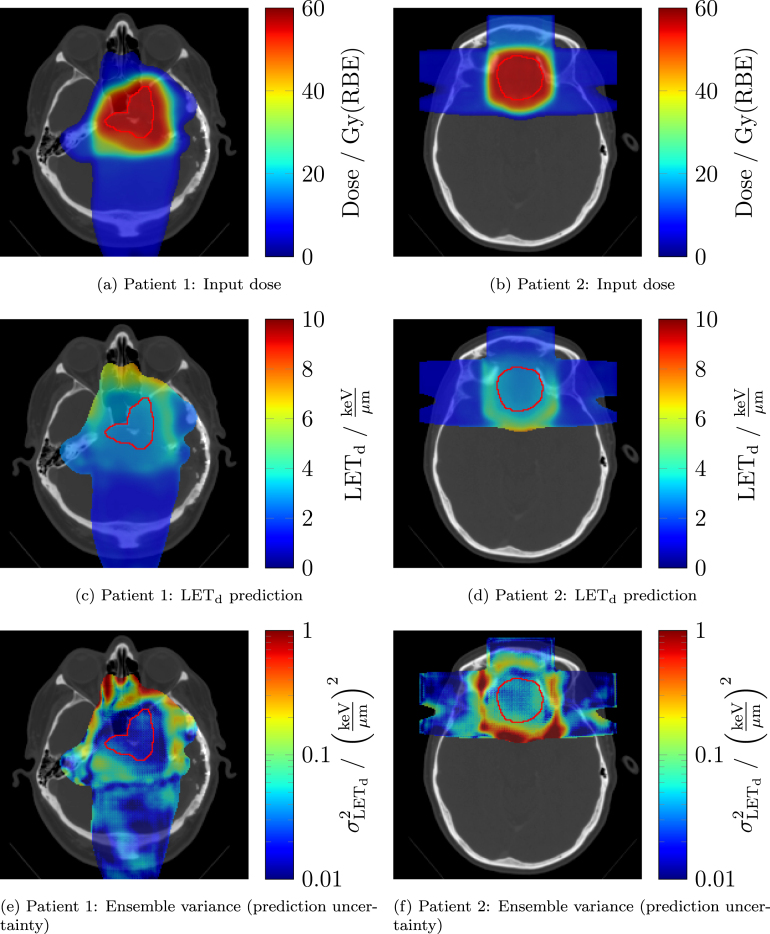
Representative examples from the MGH cohort for the combined PBS+DS model. Left column: Patient 1. Right column: Patient 2. Shown are (row 1) the planned dose distribution, (row 2) the model ${\mathrm{LET}}_{d}$ prediction, and (row 3) the predicted uncertainty map obtained via the ensemble variance method. For the MGH cohort, no reference MC ${\mathrm{LET}}_{d}$ was available. Only the relevant dose region is displayed, defined as voxels with normalised dose greater than 0.04 (dose divided by the mean dose in the CTV). Notation Gy(RBE) follows ICRU-93 definition.

For the MGH cohort, no LET${}_{d}$ reference was available, so uncertainty metrics served as the sole basis for estimating model applicability and expected performance. Fig. 4 provides representative example LET${}_{d}$ predictions and corresponding uncertainty maps obtained from the ensemble variance method of the PBS+DS model for two MGH plans. Elevated LET${}_{d}$ values and increased uncertainty were observed near distal beam edges.

**Fig. 5:**
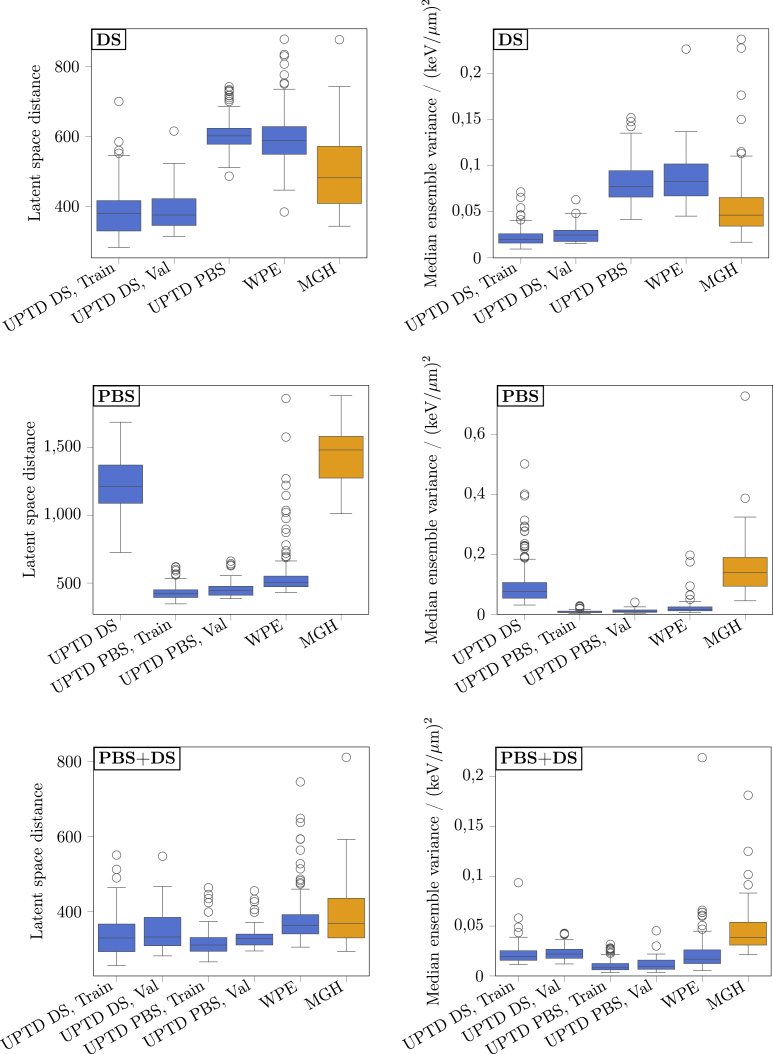
Uncertainty estimate distributions across cohorts and sets for DS, PBS, and PBS+DS models. The left column presents the results for the latent space distance, while the right column shows results for the median ensemble variance. Rows correspond to DS, PBS, and PBS+DS models. Each boxplot displays the median, the 25th and 75th percentiles (box), and the whiskers denoting the data range according to Tukey’s rule. For the MGH cohort (orange), no reference Monte Carlo (MC) LET${}_{d}$ was available, whereas for all other cohorts (blue) corresponding MC LET${}_{d}$ distributions were available. Abbreviations: Train = Training, Val = Validation.

Fig. 5 shows the distributions of latent space distance and ensemble variance for all cohorts. Across all models, the uncertainty characteristics of MGH formed a coherent pattern. For the technique-specific models, uncertainty differences between MGH and the PBS cohorts (UPTD PBS, WPE) reflected the correspondence between delivery technique and model. For the DS model, uncertainty on MGH was significantly lower than on PBS cohorts (p < 0.001, two-sided Mann–Whitney U test), indicating better expected performance on MGH than on PBS data. However, MGH showed higher uncertainty than UPTD DS for the DS model, suggesting somewhat worse performance on MGH than on UPTD DS, but still more favourable than for PBS cohorts. Based on this uncertainty analysis, we estimate that the average median VAE in the relevant dose region of the DS model on the MGH cohort is better than 0.54 keV/μm (i.e., better than the DS model performance on UPTD PBS).

For the PBS model, both uncertainty metrics were significantly higher for MGH than for PBS cohorts (p < 0.001). For the PBS+DS model, MGH exhibited significantly higher ensemble variance than all other cohorts (p < 0.001), whereas latent space distance was comparable to WPE (p = 0.523). Although this model performed robustly across all validation cohorts, the uncertainty profile indicates that its performance on MGH is likely slightly inferior or, at best, comparable to that on the other datasets. Since MGH showed the highest uncertainty among all cohorts for the PBS and the PBS+DS models, an upper bound for the prediction error could not be derived, as was possible for the DS model.

## 4 Discussion

This study extends our previously proposed CNN-based framework for predicting MC LET${}_{d}$ distributions in proton radiotherapy [25] along two axes that are critical for broader applicability: (i) broader coverage of treatment delivery techniques by adding a DS cohort and training both technique-specific and joint (PBS+DS) models, and (ii) uncertainty estimation to assess prediction reliability when reference LET${}_{d}$ is unavailable. Collectively, these advances yielded a single robust model across techniques and enabled model evaluation on external data without reference LET${}_{d}$.

We observed a pronounced degradation when applying single-technique models to the other treatment modality, which indicates limited generalisability of technique-specific models across PBS and DS and highlights that the dose–LET${}_{d}$ relationship is technique dependent.

This behaviour is consistent with fundamental differences in beam delivery and field shaping between PBS and DS. In PBS, dose is delivered by scanning narrow pencil beams with discrete energy layers and inverse planning, enabling highly conformal dose distributions. In contrast, DS employs a combination of scatterers, range modulators, apertures, and range compensators, which shape the dose through hardware-based modulation and introduce technique-specific energy spectra and distal falloff characteristics. [36], [37], [38], [39]. Additional planning factors such as optimisation strategy, beam arrangement, and target characteristics further contribute to technique- and centre-specific dose–LET${}_{d}$ relations [20], [40]. Training on both PBS and DS data thus promotes the learning of more generalisable features, enabling a single model to maintain high accuracy across techniques and cohorts.

The visual correspondence between regions of elevated ensemble variance and increased absolute prediction error, together with the moderate-to-strong correlations between uncertainty estimates and prediction error across cohorts support the use of these metrics as qualitative surrogates for model performance when reference LET${}_{d}$ data are unavailable. This provides a practical framework for assessing model applicability in external or retrospective datasets where direct validation against MC LET${}_{d}$ is not feasible. For the MGH cohort, elevated LET${}_{d}$ values at distal beam edges, together with increased uncertainty in these regions, were consistent with physical expectations and with the spatial patterns observed in cohorts with available reference LET${}_{d}$.

For the DS model, the MGH cohort exhibited higher uncertainty than the in-centre DS cohort but clearly lower uncertainty than PBS cohorts, consistent with the expectation that technique differences dominate over inter-centre variations. The same technique-related effects may also explain the uncertainty difference between the two internal UPTD cohorts (PBS and DS) in the combined PBS+DS model. Based on the uncertainty profile, we consider the DS model applicable to the MGH cohort for analyses of clinical implications related to proton RBE variability.

The present work has several limitations. First, no external DS cohort with reference MC LET${}_{d}$ was available. As a result, it was not possible to evaluate how differences in DS treatment planning strategies across institutions might influence both model performance and uncertainty, nor to disentangle potential effects of the MC engine used to generate reference LET${}_{d}$ (TOPAS vs. RayStation MC) from technique- (PBS vs. DS) and centre-specific differences.

Second, this study focused on two complementary methods for assessing epistemic uncertainty. Aleatoric uncertainty, mainly arising from MC statistical noise, was not explicitly modelled. However, because the analysis was restricted to the relevant dose region, low-dose voxels with potentially higher MC uncertainty were largely excluded, such that aleatoric uncertainty is expected to be minor in the present setting. Epistemic uncertainty is therefore expected to dominate, making ensemble variance and latent space distance suitable proxies for performance estimation. Uncertainty quantification is an active area of research in radiotherapy [32], [41], [42], [43]. Additional uncertainty estimation methods explored for dose prediction, including Bayesian neural networks, MC dropout, bagging, and evidential learning [44], [45], [46], [47], [48], may provide complementary insights into model applicability for LET${}_{d}$ prediction.

Third, uncertainty estimation cannot fully replace direct validation against reference LET${}_{d}$ data. However, it can still provide indirect evidence of model reliability in the absence of a reference and may serve as an additional safety net before model application.

In summary, the developed DL models provide accurate LET${}_{d}$ estimations for both PBS and DS treatments, and the integrated uncertainty metrics enable reliable performance assessment without reference. Overall, the results indicate that uncertainty-aware validation facilitates a safer application of LET${}_{d}$ surrogate models in retrospective analyses lacking MC reference data, thereby supporting broad investigations into variable-RBE effects in proton therapy.

## CRediT authorship contribution statement

**Aaron Kieslich:** Writing – original draft, Visualization, Software, Methodology, Investigation, Formal analysis, Data curation, Conceptualization. **Yerik Singh:** Writing – review & editing, Visualization, Software, Methodology, Investigation, Formal analysis, Data curation. **Martina Palkowitsch:** Writing – review & editing, Methodology, Investigation, Data curation, Conceptualization. **Sebastian Starke:** Writing – review & editing, Software, Methodology, Conceptualization. **Fabian Hennings:** Writing – review & editing, Methodology, Data curation. **Esther G.C. Troost:** Writing – review & editing, Resources. **Mechthild Krause:** Writing – review & editing, Resources. **Jona Bensberg:** Writing – review & editing, Software. **Armin Lühr:** Writing – review & editing, Resources. **Feline Heinzelmann:** Writing – review & editing, Resources. **Christian Bäumer:** Writing – review & editing, Resources. **Beate Timmermann:** Writing – review & editing, Resources. **Nicolas Depauw:** Writing – review & editing. **Helen A. Shih:** Writing – review & editing, Resources. **Steffen Löck:** Writing – review & editing, Supervision, Resources, Project administration, Methodology, Conceptualization.

## Declaration of Generative AI and AI-assisted technologies in the writing process

During the preparation of this work the authors used GPT-5 by OpenAI in order to improve the readability and language of the manuscript. After using this service, the authors reviewed and edited the content as needed and take full responsibility for the content of the publication.

## Data and code availability

Our training and evaluation code is publicly available at https://github.com/oncoray/deepLET_v2. The repository contains the full deep learning pipeline, including data preprocessing routines, training and inference scripts, and analysis tools for computing the reported performance and uncertainty metrics. Example configuration files and documentation are provided to facilitate reproducibility and adaptation to other clinical cohorts. The experimental outputs generated from our data analyses and trained models are publicly available at https://rodare.hzdr.de/record/4185.

## Declaration of competing interest

The authors declare the following financial interests/personal relationships which may be considered as potential competing interests: For the present study, the authors received no external financial support, neither for the study design or materials used, nor for the collection, analysis, and interpretation of data, nor for the writing of the publication.

OncoRay has a research collaboration with RaySearch Laboratories.

In the past five years, Dr. Mechthild Krause received funding for her research projects from Merck KGaA (2014–2018 for a preclinical study; 2018–2020 for a clinical study). Dr. Mechthild Krause is involved in publicly funded projects (German Federal Ministry of Education and Research) with industry participation, including Medipan GmbH, Attomol GmbH, GA Generic Assays GmbH, Gesellschaft für medizinische und wissenschaftliche genetische Analysen, Lipotype GmbH, and PolyAn GmbH (2019–2022). Dr. Krause confirms that, to the best of her knowledge, none of the above-mentioned funding sources were involved in the preparation of this paper.

Dr. Esther G. C. Troost serves as a member of the Scientific Advisory Board of IBA. She confirms that, to the best of her knowledge, the findings of this work are not related to this conflict of interest.

Dr. Steffen Löck is a Guest Editor of the virtual special issue “Analysis, expression and reporting of uncertainties in dosimetry and statistical outcome modelling in Radiation Oncology” in the journal Physics and Imaging in Radiation Oncology (PhiRO). He had no involvement in the peer-review of this article and no access to information regarding its peer-review. Full responsibility for the editorial handling and decision-making process for this manuscript was delegated to another independent journal editor.

All other authors declare no conflicts of interest.

## Appendix A Supplementary data

MMC S1 Supplementary material for uncertainty-aware deep learning LET estimation in proton therapy plans. MMC S1

## Footnotes

2 Version v8.99.30.169, RaySearch Laboratories AB, Stockholm, Sweden.

3 Version v8.99.30.101, RaySearch Laboratories AB, Stockholm, Sweden.
