# Supplementary material for: Deep learning for dose-averaged linear energy transfer estimation in pencil-beam scanning and double scattering proton radiotherapy plans with uncertainty-aware external validation
Source: Phys Imaging Radiat Oncol. 2026 May 15;39:100998. doi: 10.1016/j.phro.2026.100998 (PMC13213858; doi:10.1016/j.phro.2026.100998)
Supplement: MMC S1 — Supplementary material for uncertainty-aware deep learning LET estimation in proton therapy plans. [file mmc1.pdf]

Supplementary material: Deep learning for dose-averaged linear energy transfer estimation in pencil-beam scanning and double scattering proton radiotherapy plans with uncertainty-aware external validation

Supplementary material A: Image preprocessing

All volumetric patient data, including planned dose distributions and Monte Carlo (MC) simulated LET_d distributions, were first resampled to an isotropic voxel spacing of 1 mm using linear interpolation. For binary region-of-interest (ROI) masks, re-binarization was performed after interpolation by applying a threshold of 0.5. Each planned dose distribution was normalised on a per-plan basis by dividing all dose values by the mean dose within the clinical target volume (CTV) or, in cases with a simultaneously integrated boost (SiB), by the mean dose within the boost volume. The normalisation was performed as

$$D_\text{norm} = \frac{D}{\frac{1}{N}\sum_{i \in \text{CTV or CTV}_\text{SiB}} D_i}, \tag{S1}$$

where D and D_norm denote the original and normalised dose distributions, respectively, and N is the number of voxels within the normalisation volume. This ensures that the average dose within the CTV or CTV_SiB equals one. To mitigate the influence of voxels in low-dose regions, where high LET_d values can occur but are statistically unstable due to the limited number of contributing protons, LET_d values in voxels with a normalised dose below 0.04 were clipped to zero. The final step of preprocessing involved cropping all patient data to a fixed spatial extent of 224 × 320 × 320 voxels. The crop was centered on the bounding box of the external body contour, with its caudal limit defined by the caudal end of the brainstem contour.

Supplementary material B: Model architecture

For model development, the SegResNet architecture [1] was used. The SegResNet model is a three-dimensional convolutional neural network that adopts an encoder-decoder structure with residual blocks and skip connections. The model began with 16 convolutional filters, doubling the number of filters at each subsequent encoding level up to a maximum of 128. Between convolutional layers, a leaky ReLU activation function with alpha equal to 0.1 was applied, and instance normalisation was used to stabilize training. A ReLU function was added to the final layer to ensure non-negativity of the output $\mathrm{LET_d}$ predictions. The model was implemented using MONAI version 1.1.0 and PyTorch version 1.10.0. Training was conducted using the AdamW optimiser with a fixed learning rate of 0.0001 and a weight decay coefficient of 0.01. The loss function employed was the mean absolute error between predicted and simulated $\mathrm{LET_d}$ values on a voxel level within the region with relevant dose, defined as voxels receiving a scaled dose exceeding 0.04 after normalisation to the mean dose of the CTV, or, if present, the $\mathrm{CTV_{SiB}}$. This restriction on the region with relevant dose was used to stabilise training by excluding clinically negligible low-dose regions where sparse high-$\mathrm{LET_d}$ events can occur. Due to the large memory footprint of three-dimensional medical images, a batch size of one was used. No data augmentation was applied during training. All model training was carried out on a single Nvidia V100 GPU with 32 GB of memory.

Supplementary material C: NTCP evaluation

NTCP models

We evaluated a set of normal tissue complication probability (NTCP) models that were previously identified as sensitive to $\mathrm{LET_d}$ variations in proton therapy and were therefore suitable for assessing the clinical relevance of $\mathrm{LET_d}$ prediction accuracy [2]. The following endpoints were considered:

- **Ocular toxicity (grade ≥2, acute, ipsilateral lacrimal gland):** Risk of acute ocular toxicity of grade ≥2, modeled as a function of the maximum dose to the ipsilateral lacrimal gland [3].
- **Blindness (60 months, chiasm):** Risk of blindness within 60 months after therapy, modeled using the generalised equivalent uniform dose (gEUD, $a = 4$) to the chiasm [4].

- **Blindness (60 months, ipsilateral optic nerve):** Risk of blindness within 60 months after therapy, modeled using the gEUD ($a = 4$) to the ipsilateral optic nerve [4].
- **Memory impairment (grade ≥1, 24 months, brain):** Risk of memory impairment of grade ≥1 occurring 24 months after therapy, modeled as a function of the volume fraction of healthy brain receiving more than 35 Gy(RBE), according to the ICRU-93 definition [5].

Variable-RBE dose calculation

For all NTCP models listed above, absorbed dose and $\mathrm{LET_d}$ distributions were converted to variable RBE-weighted dose distributions using the model proposed by Wedenberg et al. [6]. A fixed α/β ratio of 2 Gy was assumed for all endpoints. The resulting variable RBE-weighted dose distributions were then used to compute the relevant dose–volume histogram (DVH) or gEUD parameters required by each NTCP model.

Patient-level risk model for contrast-enhancing brain lesions

In addition to the organ-based NTCP models, we incorporated the patient-level risk (PLR) model for the development of one or more contrast-enhancing brain lesions (CEBLs) proposed by Bahn et al. [7]. This model is derived from a voxel-wise logistic regression estimating the probability of lesion origin (POLO) as a function of absorbed dose D, $\mathrm{LET_d}$, and periventricular proximity:

$$\log\left(\frac{\mathrm{POLO}}{1-\mathrm{POLO}}\right) = b_0 + b_1\mathrm{D} + b_2(\mathrm{LET_d}\cdot\mathrm{D}) + b_3\mathrm{VP}, \tag{S2}$$

where VP is a binary indicator identifying voxels located within a 4 mm margin around the ventricular system.

This formulation implies a linear $\mathrm{LET_d}$–RBE relationship of the form

$$\mathrm{RBE}(\mathrm{LET_d}) = 1 + k\,\mathrm{LET_d}, \qquad k \approx 0.10\ \mathrm{keV^{-1}}\,\mu\mathrm{m}. \tag{S3}$$

Assuming serial tissue behaviour, voxel-wise POLO values were combined into a patient-level NTCP for the endpoint "development of one or more CEBLs":

$$\mathrm{NTCP_{CEBL}} = 1 - \prod_i \left(1 - \mathrm{POLO}_i\right), \tag{S4}$$

with i indexing all voxels considered.

Constant-RBE reference and sensitivity classification

To consistently classify patients according to their sensitivity to variable RBE effects, all NTCP endpoints were additionally evaluated under a constant RBE assumption of 1.1. This corresponds to using a constant $\mathrm{LET_d}$ value of approximately $1.06\,\mathrm{keV}/\mu$.

Patients were labeled as *sensitive* if the difference between constant- and variable-RBE NTCP predictions (based on MC $\mathrm{LET_d}$) was at least 5 percentage points. All NTCP analyses were performed on this sensitive subpopulation.

Handling of patients with multiple treatment plans

For patients treated in subseries comprising multiple plans, $\mathrm{LET_d}$ distributions were computed separately for each individual plan. The corresponding variable RBE-weighted dose distributions were then combined by voxel-wise summation. The resulting aggregate RBE-weighted dose distribution served as input to all NTCP and patient-level risk models, ensuring consistency with cumulative dose delivery.

Ventricle segmentation and post-processing

Ventricular system contours required for the CEBL risk model were generated automatically using the TotalSegmentator framework (version v2.4.0) [8, 9]. The segmentations were mapped to the voxel grid of the dose and $\mathrm{LET_d}$ data.

Automatic segmentations occasionally exhibited artifacts, such as isolated ventricle-like islands within the CTV. To address this, a topology-based post-processing procedure using 3D 6-connectivity was applied:

1. Ventricle voxels were separated into those inside and outside the CTV.
2. 3D connected components (6-neighborhood) of the entire ventricle mask were computed.
3. Components touching at least one ventricle voxel outside the CTV were identified.
4. Ventricle voxels inside the CTV belonging to components not connected to an outside-CTV region were removed.

This procedure preserved anatomically plausible ventricular continuity while eliminating isolated false-positive structures. Patients with visually unacceptable ventricle segmentations after post-processing were excluded from CEBL analysis (114/570 patients, 20 %).

Table S1: Patient characteristics of all cohorts.

Cohort	**UPTD PBS**		**UPTD DS**		**WPE**		**MGH**	
Patients / Plans	195 / 212		152 / 152		127 / 145		96 / 96	
	Median	(range)	Median	(range)	Median	(range)	Median	(range)
Age at RT (years)	56	(18–83)	50	(19–85)	42	(18–77)	46	(18–89)
Treatment fractions	30	(20–38)	30	(1–66)	30	(17–33)	25	(12–33)
CTV volume (cm^3)	148	(9–628)	146	(1–610)	74	(2–368)	28	(1–266)
largest$^{(a)}$	157	(39–361)	–		135	(20–526)	–	
smallest$^{(a)}$	79	(7–264)	–		108	(3–504)	–	
CTV mean dose (Gy(RBE)$^{(b)}$)	55	(17–72)	58	(22–74)	54	(41–61)	55	(26–72)
largest$^{(a)}$	50	(46–57)	–		52	(32–55)	–	
smallest$^{(a)}$	10	(10–57)	–		6	(2–20)	–	
	Patients	(%)	Patients	(%)	Patients	(%)	Patients	(%)
Gender (male/female)	95 / 100	(49 / 51)	87 / 65	(57 / 43)	49 / 78	(39 / 61)	38 / 58	(40 / 60)
Subseries (1/2/3)	178/17/0	(91/9/0)	152/0/0	(100/0/0)	112/12/3	(88/9/2)	96/0/0	(100/0/0)
SiB (yes/no)	118/77	(61/39)	37/115	(24/76)	0/127	(0/100)	0/96	(0/100)
Chemotherapy (yes/no)	69/126	(35/65)	64/88	(42/58)	39/88	(31/69)	22/74	(23/77)
**Tumor site**								
Astrocytoma	4	(2)	33	(22)	52	(41)	25	(26)
Glioblastoma	5	(3)	54	(36)	5	(4)	0	(0)
Glioma	4	(2)	14	(9)	24	(19)	1	(1)
Meningioma	43	(22)	22	(14)	35	(28)	38	(40)
Other	139	(71)	29	(19)	11	(9)	32	(33)
**Organs at risk**$^{(c)}$	analyzed/present	(%)	analyzed/present	(%)	analyzed/present	(%)	analyzed/present	(%)
Brain	210 / 210	(99 / 99)	142 / 142	(93 / 93)	129 / 129	(89 / 89)	96 / 96	(100 / 100)
Brain stem	199 / 212	(94 / 100)	132 / 152	(87 / 100)	133 / 145	(92 / 100)	77 / 96	(80 / 100)
Chiasm	186 / 210	(88 / 99)	118 / 150	(78 / 99)	120 / 145	(83 / 100)	62 / 88	(65 / 92)
Lacrimal gland (contra)	39 / 210	(18 / 99)	13 / 104	(9 / 68)	21 / 143	(14 / 99)	13 / 86	(14 / 90)
Lacrimal gland (ipsi)	152 / 211	(72 / 100)	63 / 111	(41 / 73)	90 / 143	(62 / 99)	35 / 87	(36 / 91)
Lens (contra)	22 / 210	(10 / 99)	11 / 147	(7 / 97)	18 / 145	(12 / 100)	4 / 84	(4 / 88)
Lens (ipsi)	92 / 212	(43 / 100)	34 / 150	(22 / 99)	51 / 145	(35 / 100)	10 / 84	(10 / 88)
Optic nerve (contra)	146 / 212	(69 / 100)	85 / 148	(56 / 97)	105 / 145	(72 / 100)	56 / 92	(58 / 96)
Optic nerve (ipsi)	172 / 212	(81 / 100)	110 / 151	(72 / 99)	116 / 145	(80 / 100)	62 / 92	(65 / 96)

Abbreviations: contra = Contralateral, CTV = Clinical Target Volume, DS = Double Scattering, ipsi = Ipsilateral, MGH = Massachusetts General Hospital, PBS = Pencil Beam Scanning, RBE = Relative Biological Effectiveness, RT = Radiotherapy, SiB = Simultaneous Integrated Boost, UPTD = University Proton Therapy Dresden, WPE = West German Proton Therapy Center Essen.

(a) For patients with multiple treatment plans: statistics computed on the patient's largest/smallest CTV subseries.

(b) According to ICRU-93 definition

(c) "Analyzed" refers to organs at risk receiving a relevant dose, defined as a maximum dose greater than 4% of the mean dose in the CTV (or CTV_{SiB} when available). "Present" refers to all organs at risk available in the dataset.

Table S2: Cohorts, training/validation splits, and their use across the three training approaches.

Cohort	Technique	Split	Patients / plans	MC LET_d reference	Role per training approach (PBS / DS / PBS+DS)
UPTD PBS	PBS	Train	153 / 167	Yes	T / V / T
UPTD PBS	PBS	Val	42 / 45	Yes	V / V / V
UPTD DS	DS	Train	119 / 119	Yes	V / T / T
UPTD DS	DS	Val	33 / 33	Yes	V / V / V
WPE	PBS		127 / 145	Yes	V / V / V
MGH	DS		96 / 96	No	U / U / U

T: used for model training within the respective training approach.
V: used for validation within the respective training approach.
U: uncertainty-only evaluation

Abbreviations: DS = Double Scattering, MGH = Massachusetts General Hospital, PBS = Pencil Beam Scanning, Train = Training, UPTD = University Proton Therapy Dresden, Val = Validation, WPE = West German Proton Therapy Center Essen.

Table S3: Model performance of the three modelling approaches (DS, PBS, PBS+DS) across the evaluated cohorts (UPTD DS, UPTD PBS, WPE) and sets (training, validation). Average median voxelwise absolute difference between Monte Carlo $\mathrm{LET_d}$ and deep learning $\mathrm{LET_d}$ in the specified regions of interest are tabled. Numbers in parentheses denote 95% confidence intervals obtained from 1000 bootstrap samples.

		Median absolute error / keV / μm										
Cohort	Set	RD	BR	BS	CTV	CH	LG-C	LG-I	L-C	L-I	ON-C	ON-I
**DS**												
UPTD DS	Train	0.13	0.12	0.38	0.07	0.36	0.55	0.44	0.52	0.51	0.97	0.44
		(0.12–0.13)	(0.10–0.12)	(0.29–0.37)	(0.06–0.07)	(0.23–0.32)	(0.19–0.63)	(0.21–0.41)	(0.20–0.66)	(0.21–0.50)	(0.41–0.79)	(0.25–0.39)
	Val	0.22	0.18	0.95	0.12	0.46	0.55	1.45	1.39	1.02	1.71	0.67
		(0.20–0.24)	(0.16–0.19)	(0.52–0.96)	(0.09–0.13)	(0.28–0.47)	(0.43–0.73)	(0.24–1.36)	(0.08–2.09)	(0.30–1.24)	(0.67–1.75)	(0.30–0.66)
UPTD PBS	Val	0.54	0.41	1.05	0.16	0.95	1.86	1.18	1.94	1.85	1.46	0.88
		(0.51–0.54)	(0.37–0.41)	(0.77–0.93)	(0.13–0.15)	(0.60–0.78)	(1.17–1.83)	(0.80–1.04)	(1.08–2.05)	(1.24–1.70)	(0.93–1.24)	(0.55–0.73)
WPE	Val	0.67	0.58	1.41	0.30	1.11	2.32	1.35	1.94	2.04	2.13	0.97
		(0.60–0.67)	(0.44–0.55)	(0.93–1.23)	(0.16–0.23)	(0.62–0.90)	(1.29–2.43)	(0.90–1.23)	(1.25–2.15)	(1.43–2.03)	(1.47–1.93)	(0.69–0.89)
**PBS**												
UPTD DS	Val	0.53	0.46	1.91	0.27	1.51	1.73	1.68	1.85	1.84	1.96	1.64
		(0.49–0.53)	(0.39–0.45)	(1.36–1.73)	(0.22–0.26)	(0.87–1.27)	(0.76–1.94)	(1.07–1.58)	(0.59–2.17)	(1.05–1.85)	(1.28–1.79)	(0.99–1.40)
UPTD PBS	Train	0.07	0.08	0.20	0.06	0.18	0.24	0.20	0.25	0.29	0.27	0.13
		(0.06–0.07)	(0.07–0.08)	(0.16–0.19)	(0.05–0.06)	(0.13–0.17)	(0.12–0.25)	(0.13–0.18)	(0.14–0.28)	(0.17–0.26)	(0.19–0.25)	(0.10–0.12)
	Val	0.12	0.15	0.37	0.10	0.32	0.52	0.39	0.84	0.87	0.50	0.39
		(0.11–0.13)	(0.13–0.16)	(0.28–0.39)	(0.08–0.10)	(0.18–0.31)	(0.21–0.58)	(0.20–0.38)	(0.45–1.14)	(0.43–0.95)	(0.27–0.50)	(0.23–0.39)
WPE	Val	0.41	0.37	0.87	0.29	0.72	0.73	0.65	0.92	1.07	1.10	0.63
		(0.31–0.38)	(0.20–0.29)	(0.50–0.72)	(0.13–0.21)	(0.34–0.55)	(0.33–0.75)	(0.38–0.57)	(0.39–0.98)	(0.56–0.98)	(0.56–0.89)	(0.38–0.53)
**PBS+DS**												
UPTD DS	Train	0.12	0.11	0.29	0.08	0.28	0.53	0.35	0.90	0.24	0.88	0.27
		(0.11–0.12)	(0.09–0.10)	(0.22–0.28)	(0.06–0.08)	(0.17–0.25)	(0.17–0.63)	(0.16–0.32)	(0.42–1.17)	(0.13–0.25)	(0.37–0.73)	(0.19–0.26)
	Val	0.22	0.17	0.84	0.16	0.55	0.92	1.35	0.84	1.33	1.89	0.65
		(0.20–0.23)	(0.15–0.18)	(0.48–0.86)	(0.10–0.17)	(0.31–0.56)	(0.55–1.33)	(0.19–1.24)	(0.22–1.28)	(0.41–1.63)	(0.62–1.82)	(0.24–0.62)
UPTD PBS	Train	0.07	0.09	0.21	0.06	0.19	0.22	0.17	0.42	0.26	0.29	0.18
		(0.07–0.07)	(0.08–0.09)	(0.16–0.19)	(0.05–0.06)	(0.14–0.18)	(0.11–0.23)	(0.12–0.16)	(0.18–0.45)	(0.17–0.24)	(0.21–0.27)	(0.13–0.16)
	Val	0.12	0.15	0.38	0.11	0.32	0.57	0.43	0.93	0.56	0.38	0.42
		(0.10–0.13)	(0.13–0.16)	(0.28–0.39)	(0.08–0.11)	(0.18–0.31)	(0.20–0.62)	(0.25–0.44)	(0.41–1.19)	(0.31–0.62)	(0.26–0.40)	(0.26–0.42)
WPE	Val	0.42	0.37	0.78	0.30	0.65	0.85	0.59	0.76	1.01	0.99	0.59
		(0.31–0.39)	(0.20–0.30)	(0.47–0.65)	(0.14–0.22)	(0.32–0.51)	(0.49–0.92)	(0.37–0.53)	(0.48–0.82)	(0.42–0.90)	(0.50–0.78)	(0.38–0.53)

Abbreviations: BR = Brain, BS = Brain stem, CH = Chiasm, CTV = Clinical Target Volume, DS = Double Scattering, L-C = Lens (Contralateral), L-I = Lens (Ipsilateral), LG-C = Lacrimal gland (Contralateral), LG-I = Lacrimal gland (Ipsilateral), ON-C = Optic nerve (Contralateral), ON-I = Optic nerve (Ipsilateral), PBS = Pencil Beam Scanning, RD = Region with relevant dose, Train = Training, UPTD = University Proton Therapy Dresden, Val = Validation, WPE = West German Proton Therapy Center Essen.

Table S4: Model performance of the three modelling approaches (DS, PBS, PBS+DS) across the evaluated cohorts (UPTD DS, UPTD PBS, WPE) and sets (training, validation). Average 98th percentile of the voxelwise absolute difference between Monte Carlo LET_d and deep learning LET_d in the specified regions of interest are tabled. Numbers in parentheses denote 95% confidence intervals obtained from 1000 bootstrap samples.

		98th percentile absolute error / keV / μm										
Cohort	Set	RD	BR	BS	CTV	CH	LG-C	LG-I	L-C	L-I	ON-C	ON-I
**DS**												
UPTD DS	Train	1.39	0.98	1.46	0.31	1.18	2.25	1.22	0.92	1.03	2.51	0.98
		(1.26–1.40)	(0.90–1.00)	(1.11–1.41)	(0.25–0.30)	(0.74–1.07)	(0.51–2.63)	(0.55–1.09)	(0.51–1.16)	(0.46–1.04)	(1.39–2.27)	(0.70–0.93)
	Val	2.34	1.77	2.84	0.48	2.25	1.44	1.98	1.76	1.41	3.54	1.55
		(1.93–2.48)	(1.52–1.89)	(1.91–3.00)	(0.36–0.51)	(1.16–2.24)	(1.00–1.71)	(0.63–1.97)	(0.18–2.68)	(0.62–1.68)	(1.81–3.77)	(0.79–1.56)
UPTD PBS	Val	2.86	2.73	2.70	0.53	1.92	2.57	1.85	2.39	2.25	2.47	1.51
		(2.75–2.88)	(2.56–2.74)	(2.33–2.63)	(0.43–0.50)	(1.32–1.67)	(1.82–2.65)	(1.41–1.71)	(1.57–2.61)	(1.69–2.17)	(1.86–2.28)	(1.16–1.40)
WPE	Val	3.03	2.98	3.34	0.73	1.89	2.88	1.92	2.58	2.50	3.37	1.89
		(2.85–3.07)	(2.67–2.99)	(2.81–3.28)	(0.55–0.68)	(1.29–1.71)	(1.79–3.14)	(1.45–1.85)	(1.77–2.84)	(1.92–2.53)	(2.64–3.27)	(1.42–1.82)
**PBS**												
UPTD DS	Val	4.25	3.85	4.46	0.81	2.88	3.29	2.82	2.43	2.25	3.71	3.08
		(3.95–4.30)	(3.53–3.88)	(3.71–4.36)	(0.70–0.80)	(2.06–2.68)	(1.46–3.87)	(2.02–2.79)	(1.07–2.79)	(1.57–2.32)	(2.85–3.61)	(2.31–2.90)
UPTD PBS	Train	0.51	0.46	0.58	0.20	0.43	0.50	0.42	0.50	0.46	0.63	0.36
		(0.49–0.52)	(0.43–0.46)	(0.52–0.58)	(0.18–0.20)	(0.34–0.41)	(0.32–0.53)	(0.31–0.39)	(0.34–0.56)	(0.35–0.45)	(0.50–0.61)	(0.29–0.35)
	Val	1.03	0.99	1.09	0.37	0.73	0.95	0.76	1.07	1.17	0.89	0.69
		(0.95–1.07)	(0.88–1.04)	(0.90–1.12)	(0.30–0.39)	(0.49–0.75)	(0.48–1.05)	(0.51–0.80)	(0.71–1.31)	(0.67–1.30)	(0.59–0.95)	(0.51–0.72)
WPE	Val	1.80	1.62	1.84	0.60	1.15	1.11	1.01	1.28	1.39	1.89	1.07
		(1.57–1.79)	(1.33–1.57)	(1.46–1.76)	(0.43–0.55)	(0.74–1.00)	(0.67–1.18)	(0.72–0.96)	(0.77–1.42)	(0.88–1.34)	(1.22–1.69)	(0.79–1.01)
**PBS+DS**												
UPTD DS	Train	1.26	0.81	1.33	0.32	1.19	2.33	1.04	1.39	0.57	2.64	0.82
		(1.14–1.27)	(0.72–0.81)	(0.94–1.26)	(0.24–0.30)	(0.62–0.99)	(0.47–2.63)	(0.46–0.91)	(0.77–1.73)	(0.31–0.59)	(1.30–2.29)	(0.57–0.77)
	Val	2.19	1.56	2.71	0.50	2.60	1.79	1.82	1.30	1.74	3.76	1.54
		(1.80–2.30)	(1.32–1.65)	(1.66–2.78)	(0.37–0.52)	(1.03–2.56)	(0.82–2.17)	(0.61–1.88)	(0.28–1.99)	(0.78–2.08)	(1.85–3.86)	(0.75–1.58)
UPTD PBS	Train	0.53	0.50	0.60	0.22	0.47	0.45	0.39	0.60	0.44	0.66	0.46
		(0.51–0.53)	(0.47–0.50)	(0.54–0.60)	(0.19–0.22)	(0.37–0.45)	(0.28–0.47)	(0.29–0.36)	(0.39–0.67)	(0.33–0.43)	(0.53–0.64)	(0.36–0.43)
	Val	1.03	1.02	1.09	0.35	0.81	0.94	0.78	1.24	0.85	0.91	0.78
		(0.96–1.07)	(0.90–1.06)	(0.91–1.14)	(0.28–0.36)	(0.48–0.81)	(0.45–1.03)	(0.57–0.82)	(0.64–1.56)	(0.52–0.93)	(0.62–0.96)	(0.54–0.80)
WPE	Val	1.76	1.54	1.72	0.62	1.04	1.44	0.94	1.21	1.28	1.69	1.08
		(1.54–1.75)	(1.26–1.50)	(1.39–1.68)	(0.46–0.57)	(0.68–0.93)	(0.86–1.56)	(0.69–0.90)	(0.91–1.34)	(0.76–1.25)	(1.19–1.57)	(0.81–1.02)

Abbreviations: BR = Brain, BS = Brain stem, CH = Chiasm, CTV = Clinical Target Volume, DS = Double Scattering, L-C = Lens (Contralateral), L-I = Lens (Ipsilateral), LG-C = Lacrimal gland (Contralateral), LG-I = Lacrimal gland (Ipsilateral), ON-C = Optic nerve (Contralateral), ON-I = Optic nerve (Ipsilateral), PBS = Pencil Beam Scanning, RD = Region with relevant dose, Train = Training, UPTD = University Proton Therapy Dresden, Val = Validation, WPE = West German Proton Therapy Center Essen.

Table S5: Normal tissue complication probability (NTCP) prediction errors from the PBS, DS, and PBS+DS models for the training and validation datasets. Reported values represent the mean absolute difference between NTCP predictions derived from Monte Carlo (MC) and deep learning (DL) LET_d. Numbers in parentheses denote 95% confidence intervals obtained from 1000 bootstrap samples. Only patients classified as sensitive ($\geq$5 percentage points difference in NTCP between constant and variable relative biological effectiveness using MC LET_d) are included. Brain lesion development results were additionally filtered to exclude cases with ventricular contour artifacts. Statistically significant differences ($p \leq 0.05$) according to the paired Wilcoxon signed-rank test are indicated with an asterisk (*).

		Absolute NTCP error				
Cohort	Set	OT	MI	Blind (ON-ipsi)	Blind (Chiasm)	BLD
**DS**						
UPTD DS	Train	0.015	0.003	0.016	0.011	0.016
		(0.011–0.018)	(0.002–0.004)	(0.011–0.022)	(0.008–0.015)	(0.013–0.019)
UPTD DS	Val	0.026	0.011	0.024	0.035	0.027
		(0.012–0.043)	(0.007–0.013)	(0.011–0.044)	(0.014–0.061)	(0.015–0.040)
UPTD PBS	Val	0.035	0.006	0.020	0.020	0.011
		(0.027–0.044)	(0.005–0.007)	(0.017–0.024)	(0.016–0.023)	(0.009–0.013)
WPE	Val	0.067	0.008	0.039	0.033	0.034
		(0.044–0.097)	(0.005–0.013)	(0.030–0.050)	(0.026–0.041)	(0.027–0.041)
**PBS**						
UPTD DS	Val	0.059	0.009	0.050	0.052	0.051
		(0.043–0.076)	(0.006–0.013)	(0.035–0.067)	(0.040–0.066)	(0.043–0.061)
UPTD PBS	Train	0.011	0.001*	0.004*	0.004*	0.004
		(0.007–0.016)	(0.001–0.001)	(0.003–0.007)	(0.004–0.005)	(0.003–0.005)
UPTD PBS	Val	0.018*	0.002*	0.006*	0.009	0.006
		(0.011–0.026)	(0.002–0.004)	(0.003–0.009)	(0.006–0.012)	(0.004–0.009)
WPE	Val	0.038*	0.005*	0.023*	0.020*	0.022*
		(0.025–0.051)	(0.004–0.008)	(0.018–0.028)	(0.016–0.025)	(0.017–0.026)
**PBS+DS**						
UPTD DS	Train	0.010*	0.001*	0.012	0.009	0.009*
		(0.006–0.015)	(0.001–0.002)	(0.008–0.016)	(0.006–0.012)	(0.007–0.011)
UPTD DS	Val	0.029*	0.011	0.022*	0.032*	0.021*
		(0.011–0.053)	(0.003–0.017)	(0.011–0.036)	(0.014–0.052)	(0.010–0.033)
UPTD PBS	Train	0.010	0.002	0.007	0.008	0.005
		(0.007–0.013)	(0.002–0.002)	(0.005–0.008)	(0.006–0.009)	(0.003–0.006)
UPTD PBS	Val	0.024*	0.002*	0.007*	0.009*	0.006*
		(0.014–0.036)	(0.002–0.004)	(0.005–0.010)	(0.006–0.012)	(0.004–0.008)
WPE	Val	0.029*	0.007	0.023*	0.021*	0.025*
		(0.019–0.040)	(0.005–0.010)	(0.018–0.028)	(0.016–0.026)	(0.020–0.031)

Abbreviations: BLD = Brain lesion development (POLO model), Blind (ON-ipsi) = Blindness 60 months after therapy based on gEUD in the ipsilateral optic nerve, Blind (Chiasm) = Blindness 60 months after therapy based on gEUD in the chiasm, DS = Double Scattering, MC = Monte Carlo, MI = Memory impairment (grade $\geq$1, 24 months, brain), NTCP = Normal Tissue Complication Probability, OT = Ocular toxicity (grade $\geq$2, acute, ipsilateral lacrimal gland), PBS = Pencil Beam Scanning, Train = Training, UPTD = University Proton Therapy Dresden, Val = Validation, WPE = West German Proton Therapy Center Essen.

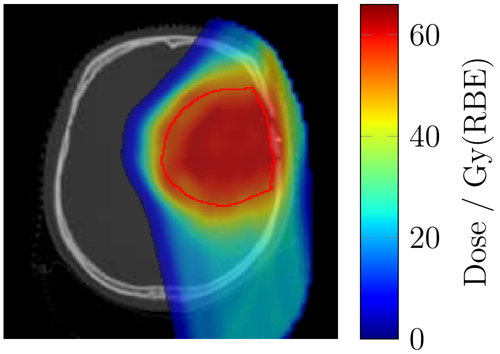

(a) Input dose

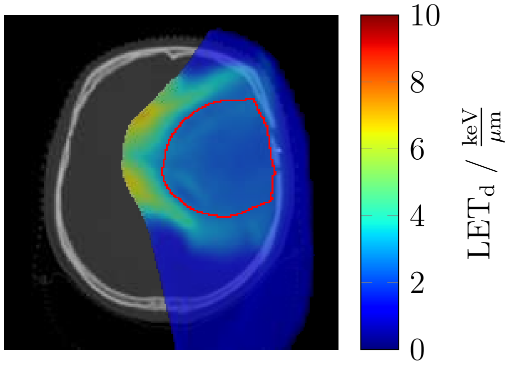

(b) Monte Carlo $\mathrm{LET_d}$

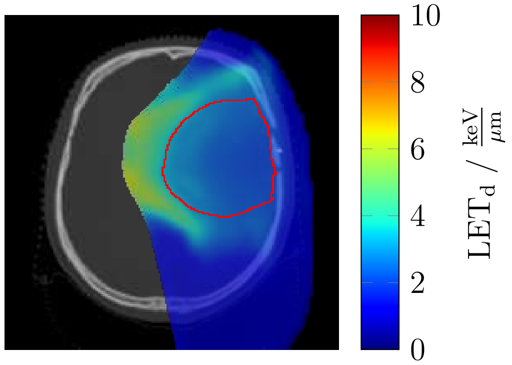

(c) $\mathrm{LET_d}$ prediction

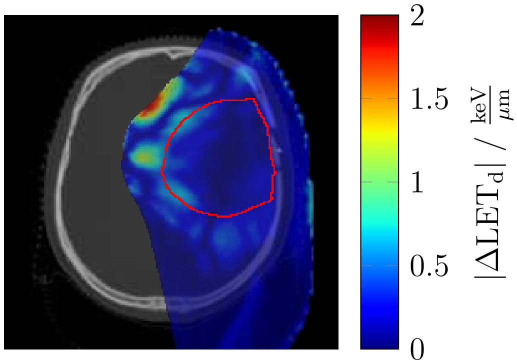

(d) Absolute $\mathrm{LET_d}$ prediction error

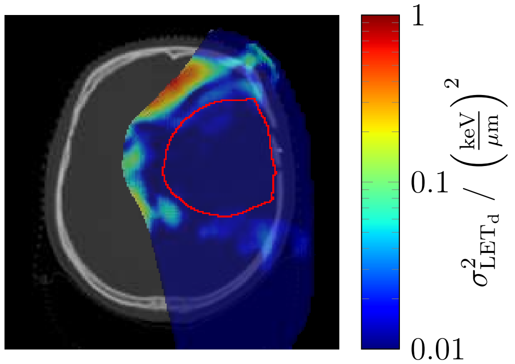

(e) Ensemble variance (Prediction uncertainty)

Figure S1: Representative example from the validation set of the UPTD PBS cohort for the combined PBS+DS model. Shown are the planned dose distribution (a), Monte Carlo reference $\mathrm{LET_d}$ (b), the corresponding model prediction (c), the voxelwise absolute $\mathrm{LET_d}$ prediction error (d), and the predicted uncertainty map from the ensemble variance method (e). The contour of the CTV is displayed in red. Only the relevant dose region is displayed, defined as voxels with normalized dose greater than 0.04 (dose divided by the mean dose in the CTV). Notation Gy(RBE) follows ICRU-93 definition.

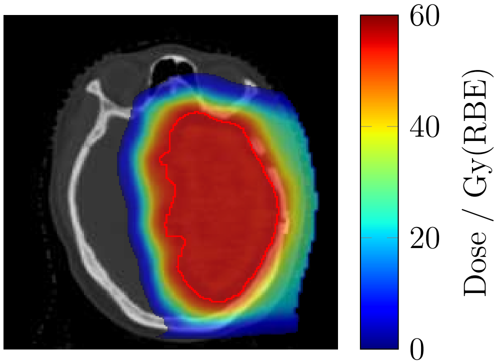

(a) Input dose

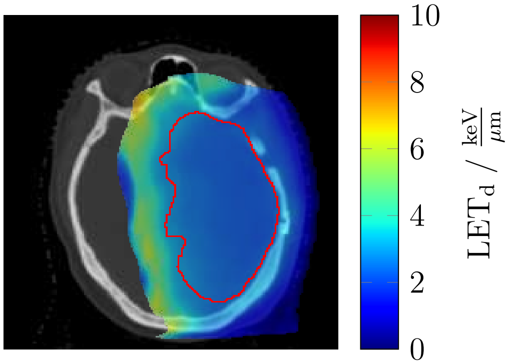

(b) Monte Carlo LET$_d$

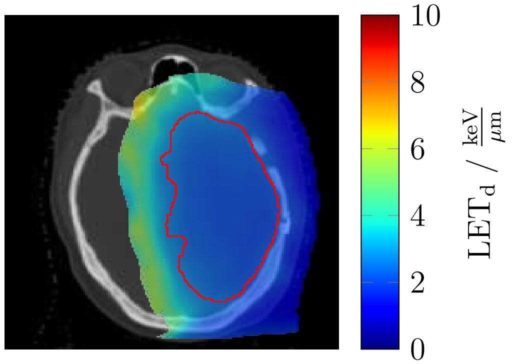

(c) LET$_d$ prediction

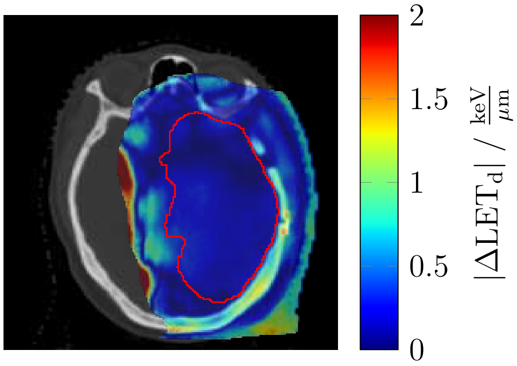

(d) Absolute LET$_d$ prediction error

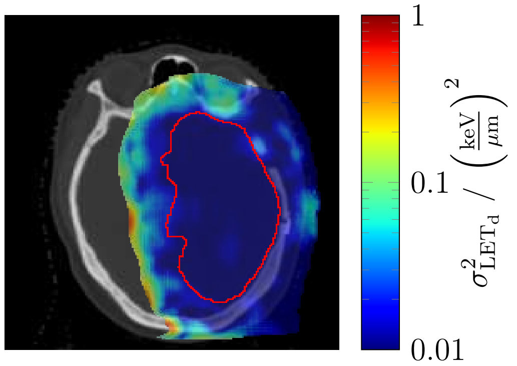

(e) Ensemble variance (Prediction uncertainty)

Figure S2: Representative example from the validation set of the WPE cohort for the combined PBS+DS model. Shown are the planned dose distribution (a), Monte Carlo reference LET$_d$ (b), the corresponding model prediction (c), the voxelwise absolute LET$_d$ prediction error (d), and the predicted uncertainty map from the ensemble variance method (e). Only the relevant dose region is displayed, defined as voxels with normalized dose greater than 0.04 (dose divided by the mean dose in the CTV). Notation Gy(RBE) follows ICRU-93 definition.

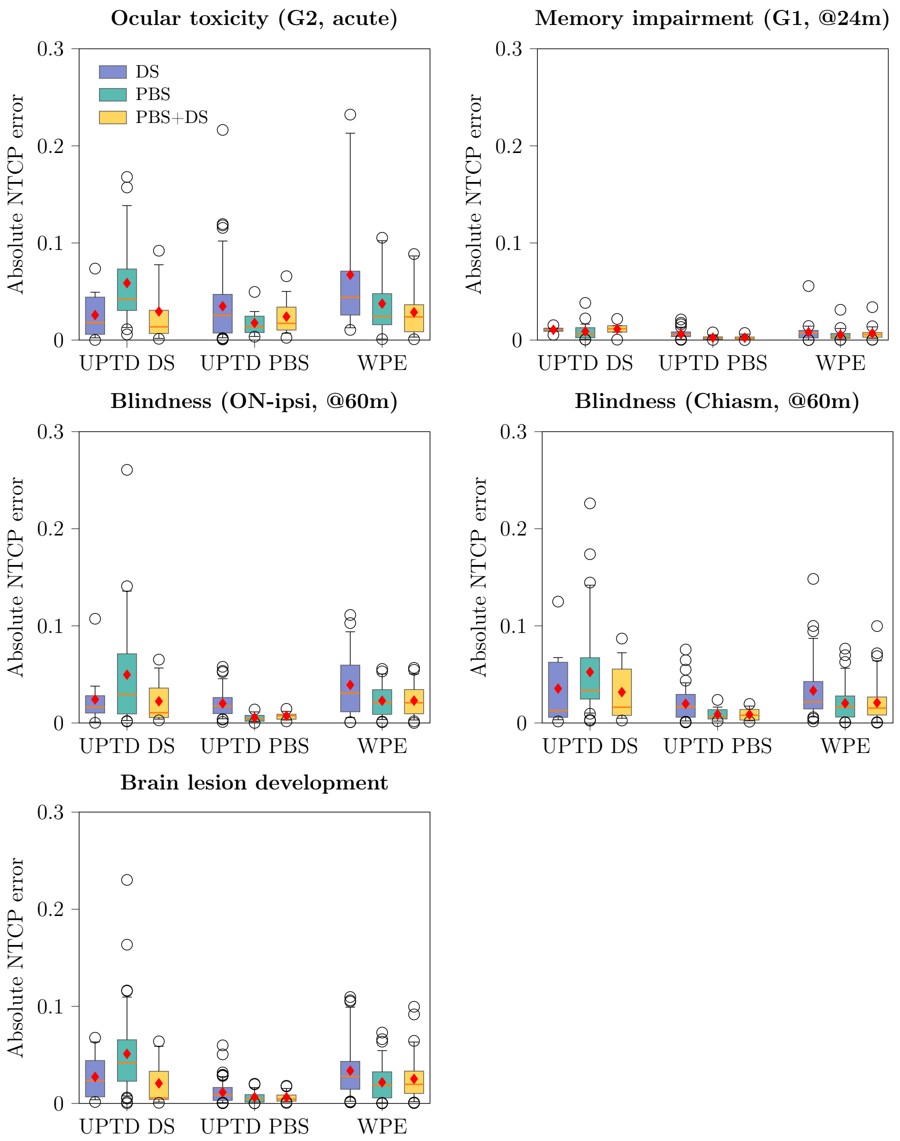

Figure S3: Absolute normal tissue complication probability (NTCP) prediction error distributions for various endpoints. The plots compare results for the different modelling approaches (DS, PBS, PBS+DS) and validation patient cohorts (UPTD PBS, UPTD DS, WPE). The error is defined as the absolute difference between NTCP prediction derived from Monte Carlo (MC) and deep learning (DL) LET_d. Only patients classified as sensitive ($\geq$ 5 percentage-point NTCP difference between constant and variable relative biological effectiveness using MC LET_d) are included. For brain lesion development, cases with uncorrectable ventricular contour artifacts were additionally excluded. Each boxplot displays the median, the 25th and 75th percentiles (box), and the whiskers denoting the data range according to Tukey's rule. Red diamonds indicate the mean absolute NTCP error within each group. Abbreviations: G = Grade, ON-ipsi = Ipsilateral optic nerve, m = Months, NTCP = Normal Tissue Complication Probability

References

[1] Myronenko A. 3D MRI Brain Tumor Segmentation Using Autoencoder Regularization. In: Crimi A, Bakas S, Kuijf H, Keyvan F, Reyes M, van Walsum T, editors. Brainlesion: Glioma, Multiple Sclerosis, Stroke and Traumatic Brain Injuries. Cham: Springer International Publishing; 2019. p. 311-20. https://doi.org/10.1007/978-3-030-11726-9_28.

[2] Starke S, Kieslich A, Palkowitsch M, Hennings F, Troost EG, Krause M, et al. A deep-learning-based surrogate model for Monte-Carlo simulations of the linear energy transfer in primary brain tumor patients treated with proton-beam radiotherapy. Phys Med Biol. 2024;69(16):165034. https://doi.org/10.1088/1361-6560/ad64b7.

[3] Batth SS, Sreeraman R, Dienes E, Beckett LA, Daly ME, Cui J, et al. Clinical-dosimetric relationship between lacrimal gland dose and ocular toxicity after intensity-modulated radiotherapy for sinonasal tumours. Br J Radiol. 2013;86(1032):20130459. https://doi.org/10.1259/bjr.20130459.

[4] Burman C, Kutcher GJ, Emami B, Goitein M. Fitting of normal tissue tolerance data to an analytic function. Int J Radiat Oncol Biol Phys. 1991;21:123-35. https://doi.org/10.1016/0360-3016(91)90172-Z.

[5] Dutz A, Lühr A, Agolli L, Bütof R, Valentini C, Troost EGC, et al. Modelling of late side-effects following cranial proton beam therapy. Radiother Oncol. 2021;157:15-23. https://doi.org/j.radonc.2021.01.004.

[6] Wedenberg M, Lind BK, Hårdemark B. A model for the relative biological effectiveness of protons: The tissue specific parameter α/β of photons is a predictor for the sensitivity to LET changes. Acta Oncol. 2013;52(3):580-8. https://doi.org/10.3109/0284186X.2012.705892.

[7] Bahn E, Bauer J, Harrabi S, Herfarth K, Debus J, Alber M. Late Contrast Enhancing Brain Lesions in Proton-Treated Patients With Low-Grade Glioma: Clinical Evidence for Increased Periventricular Sensitivity and Variable RBE. Int J Radiat Oncol Biol Phys. 2020;107:571-8. https://doi.org/10.1016/j.ijrobp.2020.03.013.

[8] Wasserthal J, Breit HC, Meyer MT, Pradella M, Hinck D, Sauter AW, et al. TotalSegmentator: robust segmentation of 104 anatomic structures

in CT images. Radiol Artif Intell. 2023;5(5):e230024. https://doi.org/10.1148/ryai.230024.

[9] Isensee F, Jaeger PF, Kohl SA, Petersen J, Maier-Hein KH. nnU-Net: a self-configuring method for deep learning-based biomedical image segmentation. Nat Methods. 2021;18(2):203-11. https://doi.org/10.1038/s41592-020-01008-z.
